# Progress and prospect of nanotechnology for cardiac fibrosis treatment

**DOI:** 10.1002/INMD.20230018

**Published:** 2023-09-05

**Authors:** Samantha L. Gaytan, Elfa Beaven, Shrikanth S. Gadad, Md Nurunnabi

**Affiliations:** ^1^ Department of Pharmaceutical Sciences School of Pharmacy The University of Texas El Paso El Paso Texas USA; ^2^ Department of Interdisciplinary Health Sciences College of Health Sciences The University of Texas El Paso El Paso Texas USA; ^3^ Department of Biomedical Engineering College of Engineering The University of Texas El Paso El Paso Texas USA; ^4^ Center of Emphasis in Cancer Department of Molecular and Translational Medicine Paul L. Foster School of Medicine Texas Tech University Health Sciences Center El Paso El Paso Texas USA; ^5^ Border Biomedical Research Center The University of Texas El Paso El Paso Texas USA

**Keywords:** cardiac fibrosis, drug delivery systems, nanomedicine, nanotechnology, pharmacological interventions

## Abstract

Cardiac fibrosis is the excessive accumulation of extracellular matrix components in the heart, leading to reduced cardiac functionality and heart failure. This review provides an overview of the therapeutic applications of nanotechnology for the treatment of cardiac fibrosis. We first delve into the fundamental pathophysiology of cardiac fibrosis, highlighting the key molecular players, including Matrix Metalloproteinases, Transforming Growth Factor‐beta, and several growth factors, cytokines, and signaling molecules. Each target presents a unique opportunity to develop targeted nano‐therapies. We then focus on recent advancements in nanotechnology and how nanoparticles can be engineered to deliver drugs or therapeutic genes. These advanced delivery approaches have shown significant potential to inhibit fibrosis‐promoting factors, thereby mitigating the fibrotic response and potentially reversing disease progression. In addition, we discuss the challenges associated with developing and translating nanotechnology‐based drug delivery systems, including ensuring biocompatibility, safety, and regulatory compliance. This review highlights how nanotechnology can bridge the gap between lab research and clinical practice for treating cardiac fibrosis.

## Graphical abstract

This review has demonstrated the advancement of nanotechnology in treating cardiac fibrosis by enabling the feature selective drug delivery to the specific cells/tissues of interest. With this cell/tissue specific delivery of therapeutic regimens, both efficacy cancer be improved, and non‐specific localization‐mediated toxicities can be controlled.

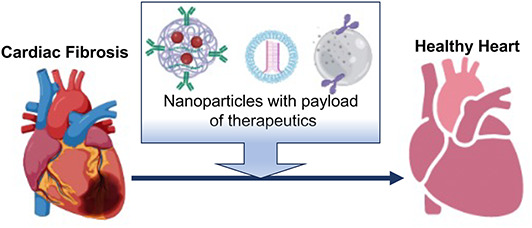

## 1 INTRODUCTION

Cardiac fibrosis and cardiovascular diseases (CVDs) impose a significant global health burden, contributing to substantial morbidity and mortality worldwide. According to recent statistics, cardiovascular disease accounts for a staggering number of deaths, estimated at 18 million annually, representing 32% of all global deaths. Cardiac fibrosis is recognized as a significant contributor to adverse outcomes and plays a pivotal role in heart disease progression among cardiovascular diseases. Recent data indicate that cardiac fibrosis is crucial in most CVDs, including heart failure and arrhythmia, which collectively affect millions worldwide.^1^

Cardiac fibrosis is characterized by the excessive accumulation of fibrotic tissue in the myocardium. This fibrous tissue results in myocardial stiffening, impaired contractility, and compromised cardiac function, making it a common pathological feature observed in conditions such as myocardial infarction, hypertension, and heart failure.^2^, ^3^ The escalating prevalence of cardiac fibrosis and its implications on global health necessitates a deeper understanding of the pathogenesis of the disease and potential therapeutic targets while underscoring the need for innovative therapeutic strategies. In this context, nanotechnology has emerged as a promising field with the potential to revolutionize cardiac fibrosis treatment by providing targeted and efficient drug delivery systems, tissue engineering approaches, and novel therapeutic strategies. Understanding the global health burden of cardiac fibrosis and cardiovascular diseases underscores the urgency and importance of advancing nanotechnology‐based interventions to address this critical health challenge.

Following cardiac injury, inflammation induces the activation of cellular growth, metalloproteinases, the proliferation of fibroblasts, and loss of myocytes, ultimately leading to scar tissue composed of a complex collagen network.^4^ The remodeling process is driven by various factors, including inflammation, oxidative stress, and fibroblast activation. These ultimately differentiate into myofibroblasts responsible for excessive ECM components and collagen production.^1^ The increasing prevalence of cardiac fibrosis poses a substantial global health burden, leading to impaired cardiac function, reduced compliance, and heightened risk of arrhythmias, ultimately resulting in significant morbidity and mortality worldwide.^5^, ^6^

## 2 CURRENT TREATMENT OPTIONS AND THEIR LIMITATIONS

Currently, the treatment of cardiac fibrosis is multifaceted, encompassing pharmacological and non‐pharmacological interventions; however, it is primarily focused on managing the symptoms and associated risk factors of underlying cardiovascular diseases.^3^ These strategies aim to control blood pressure, improve cardiac function, and address contributing lifestyle factors.^2^ For example, medications such as angiotensin‐converting enzyme inhibitors (ACE inhibitors), angiotensin II receptor blockers (ARBs), mineralocorticoid receptor antagonists (MRAs), and beta‐blockers work to reduce cardiac workload, decrease inflammation, and alleviate symptoms caused by fibrotic tissue. Pharmacological interventions are typically first‐line treatments to mitigate the progression of fibrosis. ACEIs, ARBs, and MRAs are commonly prescribed medications for patients with cardiac fibrosis.^3^, ^7^ Despite the benefits of these medications, their efficacy in reversing cardiac fibrosis remains limited. While they can help manage symptoms, they are less effective at addressing the underlying pathologies of cardiac fibrosis and thus do not address the progressiveness of fibrosis.^3^ Furthermore, these medications often have suboptimal efficacy due to their lack of targetability and specificity. They may be associated with adverse side effects, highlighting the need for innovative therapeutic options.^3^

Non‐pharmacological interventions, such as lifestyle modifications and device‐based therapies, are crucial in managing cardiac fibrosis and aim to mitigate the effects of risk factors such as hypertension, diabetes, and obesity. Lifestyle modifications include adopting regular exercise routines, dietary changes to promote heart health, and smoking cessation.^8^, ^9^ Diet and exercise are the most common lifestyle modifications in individuals with cardiac fibrosis or cardiovascular disease, and proper modifications can effectively manage the condition and improve overall heart health.^10^ Nevertheless, there may be a need for more patient education concerning these modifications, which can leave individuals uninformed about implementing these crucial changes. Additionally, the sustained commitment to these lifestyle alterations can be challenging for many patients, limiting the efficacy of these interventions. These treatments may demonstrate limited effectiveness, be associated with unfavorable side effects, or prove insufficient in halting the progression of the disease. As a result, it is imperative to develop targeted and effective therapeutic strategies that comprehensively address the manifestations of cardiac fibrosis and its fundamental etiologies to improve patient outcomes.^2^ Patients diagnosed with advanced heart failure have access to Implantable Cardioverter Defibrillators (ICDs).^8^, ^9^ CRT helps coordinate the heart's pumping action, improving efficiency.

In contrast, ICDs monitor and correct abnormal heart rhythms, reducing the risk of sudden cardiac death.^8^, ^9^ Non‐pharmacological interventions aim to reduce the impact of risk factors like hypertension, diabetes, and obesity that can worsen fibrosis, similar to medications. However, adherence to lifestyle changes can be challenging for many patients, limiting the effectiveness of these interventions.

Despite the availability of these treatment options, managing cardiac fibrosis remains a multifaceted challenge. Predominantly focused on mitigating symptoms and risk factors, many current therapeutic approaches exhibit limitations by failing to address the fundamental etiology of fibrosis.^11^ ACEIs and ARBs are among the first‐line treatments commonly prescribed to patients with cardiac fibrosis. Medications interfere with the renin‐angiotensin‐aldosterone system (RAAS), a hormone system which regulates blood pressure as well as fluid balance.^12^ By blocking the actions of angiotensin II, a potent vasoconstrictor, these medications help reduce blood pressure and alleviate strain on the heart.^12^ These medications can also be associated with various side effects. ACEIs and ARBs, for example, can cause dizziness, hyperkalemia, and renal impairment, while MRAs can lead to gynecomastia and electrolyte abnormalities.^12^

Overall, the limitations of current treatment strategies highlight the need for more effective and innovative therapies to treat cardiac fibrosis. Specifically, there is a pressing need for treatments that can directly target the pathological processes driving fibrosis, such as the overproduction of ECM components and the activation of fibroblasts. This presents an opportunity to apply novel technologies, such as nanotechnology, to develop targeted specialized therapies for cardiac fibrosis.

## 3 INNOVATIVE THERAPEUTIC STRATEGIES

Nanotechnology presents a promising avenue to advanced medicine and healthcare, particularly in drug delivery, imaging, and diagnostics.^13^, ^14^ The unique physicochemical properties of nanomaterials, including liposomes, polymeric nanoparticles, and metal‐based nanoparticles, make them excellent candidates for developing novel therapeutics designed for specific targetability, enhanced cellular uptake, and improved safety. Therefore, understanding the pathophysiology of cardiac fibrosis, including the role of various cells and molecular pathways, is crucial for developing targeted nano‐therapies that either stop fibrotic scar tissue development or reverse the process. The potential of nanotechnology in this context is further emphasized by the surge of interest in developing advanced drug delivery systems. As a result, nanotechnology‐based drug delivery systems have emerged as a promising strategy to enhance the efficacy and safety of drugs for treating a wide range of diseases, including fibrosis.^13^, ^15^, ^16^

New nanoparticle drug delivery systems use distinctive physiochemical properties, engineering encapsulation abilities, and surface modifications. These modification and formulation techniques protect drugs and biomolecules from degradation, increase circulation half‐life, improve cellular uptake, and increase the therapeutic effect.^14^, ^17^ nanoparticles can be functionalized with targeting ligands and imaging agents to enhance their specificity and allow real‐time visualization in vitro and in vivo.

Significant progress has been made in applying nanotechnology‐based drug delivery systems to treat cancer, cardiovascular disease, and neurological disorders. For instance, liposomal formulations of chemotherapy drugs have been shown to improve drug efficacy and reduce toxicity compared to traditional formulations.^16^, ^18^ Similarly, nanoparticle‐based therapies targeting the tumor microenvironment have shown promise, with several preclinical and clinical studies demonstrating encouraging results.^19^

Although significant progress has been achieved in nanotechnology, the development and translation of nanotechnology‐based drug delivery systems still need to be improved. Challenges persist in ensuring biocompatibility and safety, optimizing drug loading and release, and addressing regulatory concerns. Considering these considerations, this study aims to provide an analysis and overview of the current and potential future applications of nanotechnology in cardiac fibrosis treatment.

It will begin by understanding the underlying mechanisms and pathophysiology of cardiac fibrosis, emphasizing the molecular pathways that can be targeted using nanotechnology. Next, this review will delve into the fundamentals, types, and wide‐ranging applications of nanotechnology in medicine, focusing on its utilization in treating cardiac fibrosis. The discussion revolves around innovative approaches that address the multifaceted challenge of cardiac fibrosis, including nanoparticle‐based drug delivery systems, tissue engineering and regeneration, gene therapy, and combination therapies. By exploring studies and developments that address cardiac fibrosis and the complexity of cardiovascular disease, this review aims to provide an understanding of how nanotechnology‐based approaches can be applied to mitigate the detrimental effects of cardiac fibrosis. Moreover, this review highlights the potential of nanotechnology to improve overall patient outcomes and quality of life in the context of cardiovascular diseases. Overall, this review aims to shed light on the promising avenues for therapeutic interventions and contribute to advancing personalized medicine and effective treatment strategies for cardiac fibrosis and related conditions.

## 4 PATHOPHYSIOLOGY OF CARDIAC FIBROSIS AND POTENTIAL TARGETS FOR NANOTHERAPIES

Cardiac fibrosis is a crucial pathological process associated with cardiovascular diseases such as heart failure, hypertension, and myocardial infarction.^20^, ^21^ The fibrotic process in the heart tissue results in structural remodeling and functional impairment due to the excessive accumulation of extracellular matrix proteins. Therefore, understanding the mechanisms underlying cardiac fibrosis and its pathophysiology is fundamental for developing targeted therapeutic strategies.

**FIGURE 1:**
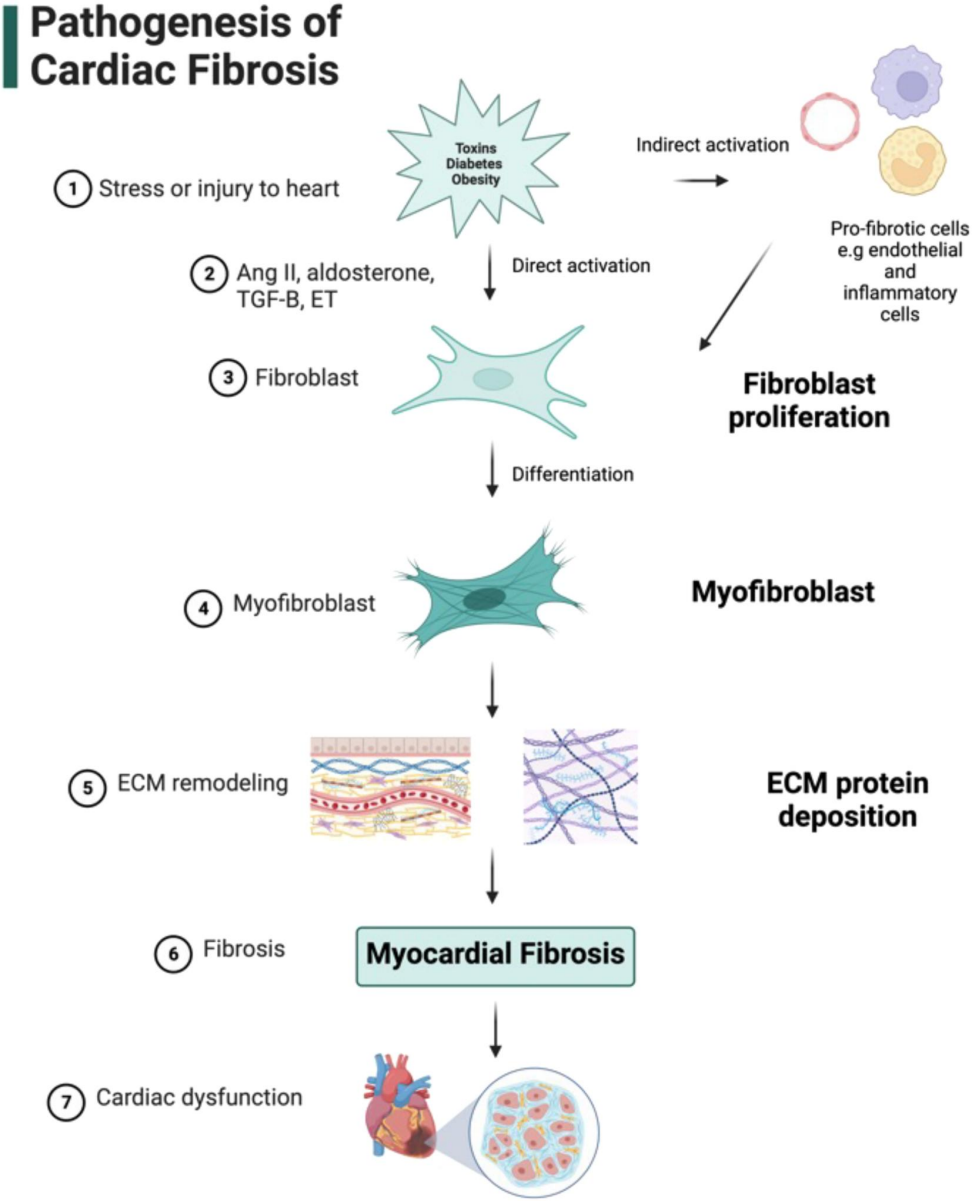
Schematic of the cardiac fibrosis development process. The process begins with stress or injury to the heart (1), triggering the release of angiotensin II, aldosterone, and transforming growth factor‐beta (TGF‐β) (2). These factors induce the differentiation of fibroblasts (3) into myofibroblasts (4), which play a crucial role in extracellular matrix (ECM) remodeling (5). ECM remodeling ultimately leads to myocardial fibrosis (6), which can cause cardiac dysfunction (7). This schematic highlights the important steps and factors involved in the development of cardiac fibrosis. Created with BioRender.com.

The normal healthy heart is composed of about 75% cardiomyocytes surrounded by fibrillar collagen that aids in transmitting contractile force.^6^ Vascular endothelial cells, immune cells, smooth muscle cells, and pericytes also comprise the cardiac interstitium. Cardiac fibroblasts, similarly abundant within the cardiac tissue, are responsible for maintaining structural integrity, regulating collagen turnover, and synthesizing and degrading ECM proteins. In response to myocardial injury, inflammatory cells and growth factors are released, binding to cell receptors to initiate a cascade of fibrogenic intracellular and extracellular signaling. Consequently, fibroblasts will transition into myofibroblasts at the injury site, prompting the fibrotic scar‐forming process (Figure 1).^22^

The pathological process of cardiac fibrosis involves several vital molecules and pathways which constitute potential targets for nanotechnology‐based treatments. In response to stress factors and injury, such as increased workload, cardiac remodeling processes are initiated, including cardiac hypertrophy, altered cardiac gene expression, extracellular matrix (ECM) deposition, and eventually fibrosis. The renin‐angiotensin system (RAS) and transforming growth factor‐ β (TGF‐β1) are well‐known contributors to the progression of cardiac pathologies, including cardiac fibrosis.^2^, ^21^

### 4.1 Matrix Metalloproteinases (MMPs)

Matrix Metalloproteinases (MMPs) are a group of molecules involved in the pathophysiology of cardiac fibrosis. MMPs comprise a family of enzymes that play a significant role in tissue remodeling by degrading extracellular matrix (ECM) components.^23^ In a healthy state, the equilibrium between the activities of MMPs and their tissue inhibitors (TIMPs) allows for regular tissue remodeling and repair.^6^ However, this balance is disrupted in cardiac fibrosis, leading to excessive ECM deposition and myocardial stiffening.^24^ ^,^ ^25^

### 4.2 Transforming growth factor‐beta (TGF‐β) and other growth factors

TGF‐β is another crucial molecular player in the fibrotic processes. This cytokine is instrumental in driving the activation of fibroblasts into myofibroblasts, the cells responsible for excessive ECM production in cardiac fibrosis.^21^, ^26^ Under pathological conditions, TGF‐β is highly expressed in diseased hearts and is responsible for driving many of the fibrotic signaling pathways. The TGF‐β signaling pathway is activated by its attachment to TGF‐β surface receptors which phosphorylate Smad2/Smad3 downstream mediators. However, it can also stimulate a cascade of non‐Smad signaling pathways. This subsequent activation leads to downstream signaling events responsible for driving inflammatory pathways, cellular activation, proliferation, fibroblast trans‐differentiation, collagen synthesis, deposition, and α‐SMA expression, all leading to uncontrolled fibrosis progression.^27^ For instance, TGF β‐1 released from cardiac fibroblasts mediated angiotensin II‐induced cardiac hypertrophy. Additionally, the upregulation of angiotensin II and TGF β‐1 is associated with fibrosis in the heart.^7^ This association is one of the many reasons RAS and TGF β‐1 signaling pathways are crucial targets for developing pharmaceutical interventions to attenuate fibrosis progression.

In addition to MMPs and TGF‐β, several other molecular targets have been identified in cardiac fibrosis, such as connective tissue growth factor (CTGF), platelet‐derived growth factor (PDGF), and various interleukins. These targets offer a broad landscape for the development of targeted nano‐therapies. Nanoparticles can be engineered to carry drugs or gene therapy vectors that inhibit the action of these fibrosis‐promoting factors. For instance, nanoparticles could be designed to deliver therapies directly to the heart tissue, reducing systemic side effects and improving treatment efficacy. In addition, certain research efforts in this direction, which will be discussed in more detail later in this review, have shown promising results in preclinical models of cardiac fibrosis. As such, nanotechnology‐based therapies hold significant promise for providing more effective treatments for this disease.

### 4.3 Cellular targets

Cardiomyocyte death, pressure overload, and myocardial inflammation can activate fibrotic wound healing. Various cell types are involved in fibrotic pathways, including fibroblasts, macrophages, mast cells, lymphocytes, cardiomyocytes, and vascular cells.^5^ The involvement of each cell type depends on the cause of the initial injury; however, the fibroblast to myofibroblast transition is a critical event that drives the continued deposition of scar tissue in the myocardium. Resident cardiac fibroblasts in a healthy heart do not secrete large number of ECM proteins or collagen; however, after injury, releasing growth factors and inflammatory cytokines promotes the mechanical stress on the fibroblasts inducing a phenotypical change.^28^ TGF‐β activation promotes α‐SMA transcription, a key hallmark of the trans‐differentiation into myofibroblasts.^29^ Myofibroblast cells are responsible for the synthesis and deposition of collagen.

Monocytes and macrophages are responsible for releasing large number of pro‐inflammatory cytokines such as Interleukin (IL)‐1β, Tumor Necrosis Factor (TNF)‐α, and IL‐6 and profibrotic growth factors such as TGF‐β, PDGFs, and FGFs. These cells start the initial activation of the wound‐healing process; however, due to chronic stimulation, they continue driving the fibrotic process.

### 4.4 Extracellular matrix barrier

The abundant extracellular matrix (ECM) consistently poses a challenge to effective therapy, necessitating the successful penetration of nanoparticles for optimal outcomes. Various strategies have been developed to overcome these barriers. One approach involves surface modification of nanoparticles with ECM‐degrading enzymes or peptides, such as MMPs, which can facilitate ECM remodeling and enhance nanoparticle penetration.^30^ By incorporating MMPs on the surface of nanoparticles, they can actively degrade the ECM components, allowing deeper tissue penetration and improved therapeutic efficacy.

Another strategy involves using stimuli‐responsive nanoparticles that can undergo size or charge changes in response to specific triggers in the ECM microenvironment. For example, pH‐responsive nanoparticles based on poly(ethylene glycol)‐poly(β–amino ester) copolymers can shrink or disassemble in the acidic tumor microenvironment, enabling better penetration and drug release.^31^ These nanoparticles can take advantage of the acidic pH characteristic of the ECM to enhance their penetration and deliver therapeutic agents precisely to the target site.

These engineering strategies highlight the innovative approaches employed to overcome the ECM barrier and enhance the effectiveness of nanoparticle‐based therapies. By tailoring the surface properties and responsiveness of nanoparticles, researchers are advancing the field of nanomedicine and improving the delivery of therapeutics to treat diseases such as cardiac fibrosis.

## 5 NANOTECHNOLOGY IN MEDICINE

Nanotechnology has shown immense potential and emerged as a formidable strategy for revolutionizing medicine and healthcare. It offers innovative solutions across various medical applications, including drug delivery, imaging, and diagnostics.^16^, ^32^ The inherent advantages of nanomaterials, such as their high surface area‐to‐volume ratio, tunable size, and versatile surface chemistry, enable the development of advanced drug delivery systems. These systems can address the complex challenges encountered in medical research and clinical practice, enhancing existing therapeutics efficacy, specificity, and safety.^13^, ^16^, ^33^ For instance, nanocarriers can improve drug bioavailability, solubility, and stability while also allowing for targeted and controlled release, thus minimizing the side effects of conventional drug administration. One such example is the development of targeted nanoparticles carrying anti‐fibrotic agents, such as pirfenidone or tranilast, which can efficiently deliver these drugs to fibroblasts and myofibroblasts, inhibiting excessive extracellular matrix (ECM) production and reducing cardiac fibrosis.^19^ Additionally, nanoparticle‐based gene therapy has shown potential in modulating the expression of critical fibrosis‐related genes, such as transforming growth factor‐beta (TGF‐β) and connective tissue growth factor (CTGF), thereby attenuating the pathological fibrotic response.^34^ Researchers have also explored using stimuli‐responsive nanoparticles in a broader scope of drug delivery. Specialized nanoparticles are engineered to release therapeutics when environmental triggers such as changes in pH or temperature are detected. Although this strategy has not been explicitly applied to cardiac fibrosis, it holds promise for ensuring targeted and controlled drug release at the site of fibrotic lesions, thereby enhancing treatment efficacy and minimizing side effects.^19^ ^,^ ^35^ Nanomaterials can be employed in diagnostic platforms such as biosensors, providing rapid, sensitive, and cost‐effective solutions for disease detection.^33^, ^36^

Nanoscale devices and sensors offer potential breakthroughs in the early detection and monitoring of diseases, paving the way for more personalized and precise medicine. In the field of medical imaging, nanotechnology‐based contrast agents have improved the resolution and specificity of imaging modalities such as magnetic resonance imaging (MRI), computed tomography (CT), and positron emission tomography (PET).^37^ Furthermore, nanomedicine holds tremendous potential for enhancing patient outcomes and transforming healthcare in many diseases and disorders including cancer, cardiovascular diseases, and neurological disorders.^14^ Although these approaches are still in the early stages of development, the potential of nanotechnology to transform cardiac fibrosis treatment is evident. It continues to drive further exploration and innovation.^35^

## 6 NANOTECHNOLOGY‐BASED DRUG DELIVERY SYSTEMS: A TRANSFORMATIVE APPROACH TO THERAPEUTICS

In recent years, researchers have explored various approaches utilizing nanotechnology to address the challenges associated with treating cardiac fibrosis. These approaches primarily involve designing and engineering nanoscale drug delivery systems that selectively target fibrotic cardiac tissue, improve drug bioavailability, and minimize off‐target effects. Nanotechnology‐based drug delivery systems leverage the unique physicochemical properties of nanomaterials to enhance the therapeutic potential of drugs.^38^ Encapsulation, binding, or conjugation of drugs into or onto nanoparticles enables controlled drug release, enhanced drug stability, and improved biodistribution.^14^ Nanotechnology‐based drug delivery systems offer a transformative approach to therapeutics capitalizing on nanoscale materials unique properties to enhance drug treatment effectiveness and safety.^13^ These systems primarily consist of nanoparticles engineered to carry drugs, protect them from degradation, and release them controlled at the targeted site of action.

Nanoparticles used for drug delivery can take various forms including liposomes, polymeric nanoparticles, dendrimers, micelles, and inorganic nanoparticles, each with distinctive advantages and potential applications.^16^ These nanoparticle types can be further functionalized with targeting ligands, such as antibodies or peptides, to enhance their specificity toward certain cell types or tissues. Additionally, the surface of nanoparticles can be modified to improve their stability, prolong circulation time, and evade the immune system. By optimizing these drug delivery capabilities, off‐target effects are reduced, ultimately enhancing therapeutic outcomes.^34^, ^37^

Consider the potential application of functionalized nanoparticles in the treatment of cardiac fibrosis. Targeting ligands, such as antibodies or peptides, can be attached to nanoparticles to selectively recognize and bind to fibrotic tissues in the heart. This targeted approach minimizes unintended effects on healthy tissues. It maximizes the delivery of therapeutic agents precisely to the affected areas, offering a promising strategy to combat the intricacy of cardiac fibrosis.

### 6.1 Liposomes

Liposomes, composed of phospholipid bilayers, were among the first nanocarriers developed for drug delivery.^39^ Liposomes are vesicular structures consisting of lipid bilayers that can encapsulate hydrophilic and hydrophobic drugs, enhancing their stability and bioavailability.^16^, ^39^ Liposomes provide versatility in drug delivery, reduce off‐target effects, and provide controlled release of encapsulated drugs.^14^ They have been extensively used in the clinical setting, with Doxil®, a liposomal formulation of the chemotherapeutic drug doxorubicin, the first FDA‐approved nano‐drug.^15^ Notably, the benefits of liposomal drug delivery are not confined to oncology but also to cardiology. In addition, the unique properties of liposomes can be harnessed to develop targeted therapies for a range of cardiovascular diseases including cardiac fibrosis.

Clinical trials investigating antifibrotic therapies have demonstrated modest effects but were not designed to reverse established fibrosis. However, Rurik et al. have shown the versatility of liposomes in improving cardiac function after heart injury. Previous work has highlighted that eliminating activated fibroblasts in mouse models reduces cardiac fibrosis. In this context, this group developed an mRNA‐encapsulated lipid nanoparticle capable of producing CAR T cells after injection, eliminating the need for ex vivo T‐cell extraction from patients.^40^

**FIGURE 2:**
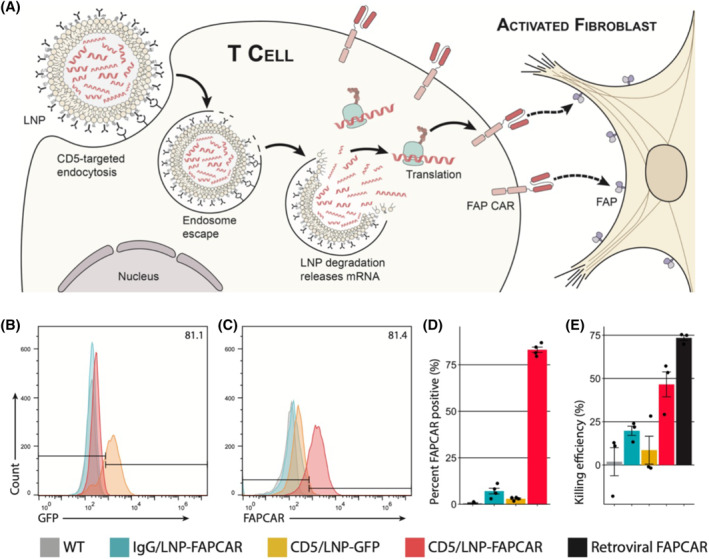
Schematic representation of CD‐5 targeted LNP. (A) Illustration depicting the process by which the CD‐5 antibody targets the T cells releases mRNA and translates expression of FAPCAR to target activated fibroblasts. (B) GFP expression after 48 h of incubation. (C) FAPCAR expression after 48 h of incubation. (D) Quantitative percentage of FAPCAR‐positive expression. (E) Percentage of killing efficiency. CD‐5 is a cell surface antigen expressed on T cells; CAR, chimeric antigen receptor; FAP, fibroblast activation protein; FAPCAR, a CAR T cell expressing FAP; GFP, green fluoresencent protein. Reproduced with permission.^40^ Copyright 2022, American Association for the Advancement of Science.

The lipid nanoparticle (LNP) surface was modified with CD‐5 antibodies to actively target T cells, given their expression in these cells. This modification allowed for specific delivery of the mRNA therapeutic. Upon attachment of the antibody to a receptor, the lipid structure of LNP was readily endocytosed by the T cells, enabling the release and cytoplasmic transcription of the mRNA. The nucleoside‐modified mRNA was sequenced with a chimeric antigen receptor (CAR) design targeting fibroblast activating protein (FAP), transforming host T cells into transient CAR T cells. Histological analysis of this study revealed a significant improvement in collagen deposition within the ECM when comparing the CD‐5 targeted lipid nanoparticle encapsulating mRNA to the control groups. In certain instances, the ECM in the targeted LNP group was indistinguishable from the healthy control group. The study demonstrated that lipid nanoparticles increased the therapeutic effect and facilitated the reversal of established fibrosis. Surface modification of the LNP with a targeting antibody enhanced cellular uptake, shown by an 83% expression of FAPCAR in host T cells compared to 7% following exposure to nontargeted T‐cells (Figure 2).^40^

### 6.2 Polymeric nanoparticles

Due to their tunable properties and biocompatibility, polymeric nanoparticles are widely utilized in various fields including drug delivery, imaging, and therapeutic development. These nanoparticles are formed from polymers such as polylactic acid (PLA), polyglycolic acid (PGA), and copolymers. The choice of polymer can be tailored to achieve specific properties such as biocompatibility, biodegradability, or the ability to respond to environmental stimuli like pH or temperature. These materials offer a high degree of flexibility in particle size, shape, and surface properties, enabling the tuning of drug release kinetics and biodistribution in therapeutic applications.

One of the critical advantages of polymeric nanoparticles is their capacity for encapsulation and controlled release of drugs. The encapsulation of drugs within polymeric nanoparticles can help to protect them from degradation within the body, and the release can be controlled through the design of the polymer matrix. In addition, they can encapsulate a broad range of therapeutic agents, including small‐molecule drugs, proteins, peptides, and nucleic acids.^14^ Additionally, the surface of polymeric nanoparticles can be modified with targeting ligands to direct them to specific cells or tissues, improve cellular uptake and enhance the overall therapeutic efficacy.

A notable example is in a study by Yao et al., who investigated the potential of polymeric nanoparticles in treating atherosclerosis. The researchers developed a mechanically responsive and inflammatory macrophage‐targeted hydrogel carrier for the management of atherosclerosis. The hydrogel carrier was designed using a polymeric framework that could respond to mechanical stimuli mimicking the dynamic environment of atherosclerotic plaques. The carrier also exhibited dual sensitivity to pH and temperature changes enabling triggered drug release at the site of inflammation. In addition, the hydrogel carrier specifically targeted inflammatory macrophages, critical players in atherosclerotic plaque development. This study demonstrated that this innovative polymeric hydrogel drug carrier effectively reduced plaque inflammation and promoted plaque stabilization in a preclinical model of atherosclerosis.^41^

Although this study was not focused on cardiac fibrosis, atherosclerosis, and cardiac fibrosis are pathological processes that significantly contribute to cardiovascular disease and are interlinked in several ways. First, atherosclerosis is characterized by the buildup of plaques in the arteries, leading to narrowing and hardening of these vessels. This impedes blood flow and can lead to cardiovascular events such as myocardial infarction. Following a myocardial infarction, the body's repair mechanisms initiate a wound‐healing response, which includes inflammation and fibrosis. Fibrosis in the heart can occur after myocardial infarction as the body attempts to replace the damaged heart tissue with scar tissue. However, this scar tissue lacks the contractile properties of healthy heart tissue, leading to impaired heart function and, potentially, heart failure. Moreover, it is worth noting that chronic inflammation, a key component in the progression of atherosclerosis, can also directly induce fibrosis.

**FIGURE 3:**
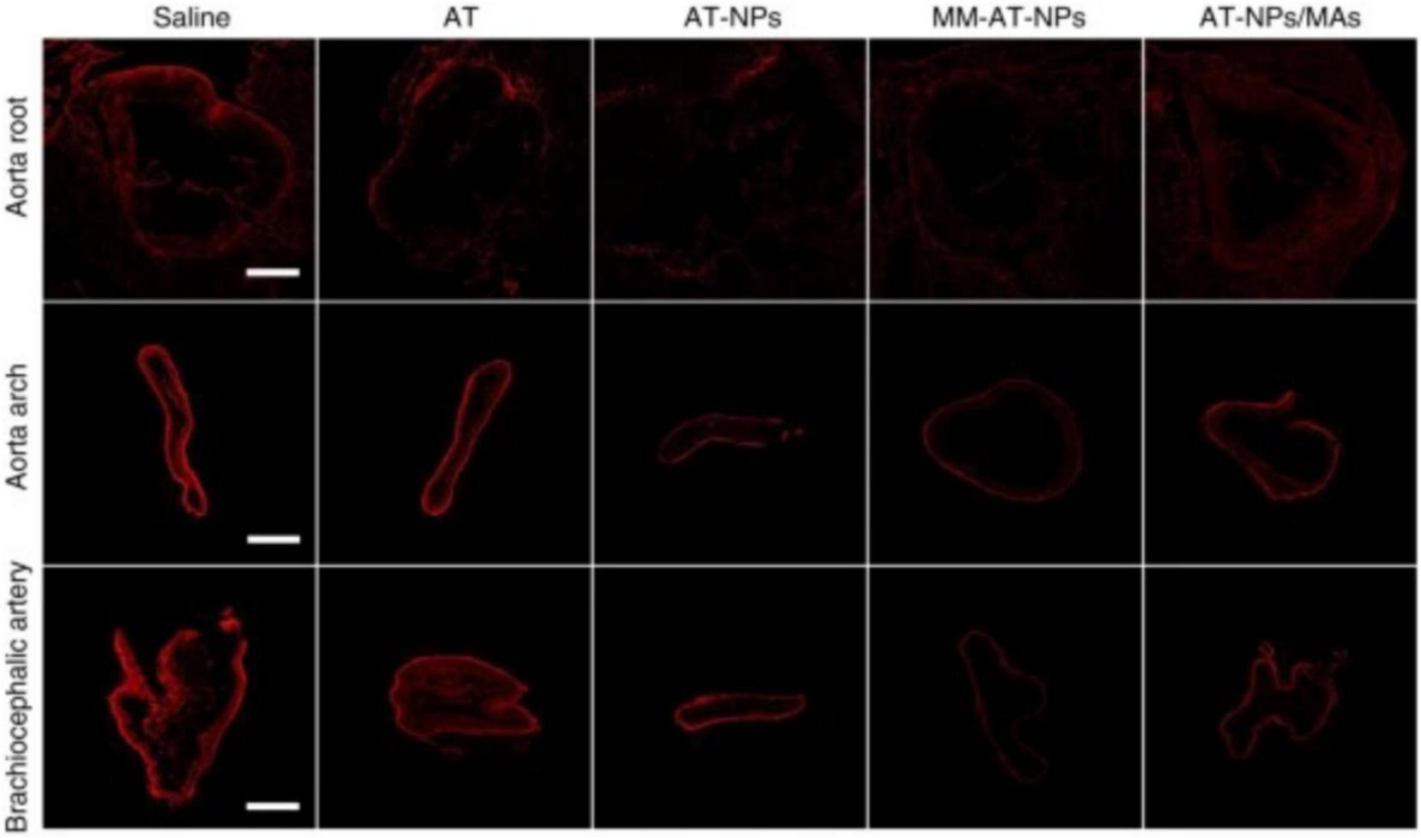
Evaluation of anti‐atherosclerotic actions by MM‐AT‐NPs. DHE‐stained sections of the aorta root, aorta arch, and brachiocephalic artery from atherosclerotic mice treated with different formulations. Red fluorescence represents the production of ROS. Stained aortic tissues indicated that AT‐NPs/MAs significantly reduced the production of ROS compared to the saline group, showing the ability of this particle to attenuate oxidative stress among atherosclerotic mice. AT, atorvastatin; AT‐NPs/MAs, MM‐AT‐NPs and AT‐NPs internalized inside macrophages; DHE, dihydroethidium; MM‐AT‐NP, macrophage membrane coated AT loaded nanoparticle; NPs, nanoparticles; ROS, reactive oxygen species. Reproduced with permission.^38^ Copyright 2020, Springer Nature.

Another study by Gao et al. focused on macrophage‐biomimetic nanoparticles addressing the proinflammatory nature of atherosclerotic plaques.^38^ These nanoparticles were engineered using a polymeric core encapsulating anti‐inflammatory drug and a surface coating derived from macrophage membranes. The macrophage membrane coating mimicked the natural targeting properties of macrophages, enabling specific accumulation at the sites of inflammation within atherosclerotic plaques. The nanoparticles demonstrated the ability to sequester proinflammatory cytokines, reducing local inflammation and promoting plaque stabilization Figure 3. The results of this study highlighted the potential of polymeric nanoparticles in atherosclerosis treatment by combining targeted pharmacotherapy with the biomimetic properties of macrophages, providing a novel approach for addressing the inflammatory processes associated with the disease.^38^

In a study by Nakashiro et al., the authors aimed to explore the potential of targeted nanoparticle‐based therapies for atherosclerotic plaques, a key factor contributing to cardiac fibrosis.^42^ They developed nanoparticles loaded with the statin drug pitavastatin, a medication commonly used to lower cholesterol levels. The goal was to have these nanoparticles target and concentrate the statin in atherosclerotic plaques, improving therapeutic efficacy and minimizing systemic side effects. The nanoparticles were explicitly designed to be taken up by inflammatory monocytes, a type of white blood cell involved in the immune response and typically found in high numbers at sites of inflammation. Once inside the monocytes, the nanoparticles are transported to the plaque site, releasing the pitavastatin. One of the primary outcomes monitored in this study was the expression of Matrix Metalloproteinase‐9 (MMP‐9), an enzyme involved in ECM remodeling and associated with plaque instability and rupture. In addition, the nanoparticle‐mediated delivery of pitavastatin led to a significant reduction in MMP‐9 expression, contributing to plaque stabilization.

These studies demonstrated nanotechnology's promising potential for treating cardiovascular diseases. Furthermore, they highlight that the relationship between atherosclerosis and cardiac fibrosis underscores the complexity of cardiovascular disease and the need for therapies that can address multiple aspects of this condition.

Polymeric nanoparticles hold immense potential in nanomedicine, offering controlled and targeted drug delivery, tissue regeneration, and imaging capabilities. Studies demonstrating their applications in cardiovascular disease treatment highlight their effectiveness in site‐specific drug delivery and therapeutic outcomes. With further advancements and optimization, polymeric nanoparticles are poised to revolutionize the management of cardiovascular diseases and improve patient outcomes.

### 6.3 Metallic nanoparticles

The limitations of current treatments for cardiac fibrosis are primarily focused on symptomatic relief rather than the regression of fibrosis. This highlights the need for innovative treatment approaches to reverse cardiac fibrosis. A promising method to achieve this involves using functionalized metallic nanoparticles that can improve the targetability and specificity of fibrosis treatments. Additionally, inorganic nanoparticles, such as gold nanoparticles, quantum dots, and magnetic nanoparticles, have gained interest due to their unique optical, electrical, and magnetic properties, which can be harnessed for imaging, thermal therapy, and controlled drug release.^18^ Metallic nanoparticles, such as gold and silver, are increasingly used in drug delivery due to their unique optical, electronic, and thermal properties. They can be functionalized with various molecules including drugs, proteins, and DNA, enabling multiple applications in targeted drug delivery, gene therapy, and theranostics.^37^

Cardiac hypertrophy is a progressive disease resulting from chronic pressure overload and can eventually evolve into cardiac fibrosis. Melatonin, a molecule produced by the pineal gland, has been shown in prior research to possess anti‐fibrotic properties in the heart, achieved by inhibiting TGF‐ β ‐SMAD3 signaling pathway and protecting the myocardium from diastolic chamber stiffness. However, the conventional oral administration of melatonin limits its therapeutic potential as an anti‐fibrotic agent.

In this context, a study conducted by Zhao et al. utilized a combination of cardiac homing peptide (CHP) and superparamagnetic iron oxide nanoparticles (SPIONs) for co‐targeted delivery of melatonin specifically to the hypertrophied heart tissue, aided by an external magnetic field.^43^ They developed a nanoplatform that employed CHP‐functionalized SPIONs to deliver melatonin with enhanced specificity to hypertrophied myocardial tissue. While SPIONs possess strong magnetic properties enabling them to be guided to the target, their accuracy in cellular targeting is limited. The addition of CHP to the platform was shown to address this issue, enhancing the specificity of delivery to diseased cardiac tissue. The results from ex vivo imaging and histological staining demonstrated a reduction in cardiac hypertrophy and fibrosis, indicating that the dual‐action functionalized nanoparticle increased the cellular uptake of melatonin. Further, echocardiography revealed a restoration of cardiac function, supporting the efficacy of this nanotechnology‐based approach for treating cardiac fibrosis.^43^

### 6.4 Dendrimers and micelles

Dendrimers and micelles, although less frequently used, also offer unique opportunities for drug delivery with their highly branched structures and self‐assembling properties, respectively.

Dendrimers, highly branched macromolecules with a well‐defined architecture, have emerged as promising nanocarriers for targeted drug delivery in cardiovascular disease treatment (Smith et al., 2010). They have gained significant attention in nanomedicine due to their unique properties and versatility, such as structure, high surface functionality, and tunable properties. These unique characteristics of dendrimers allow for precise control over their size, surface functionality, and drug‐loading capacity, making them promising candidates for targeted drug delivery and therapeutic applications.^41^, ^44^ These nanocarriers also offer advantages such as controlled drug release, enhanced stability, prolonged circulation time, and the ability to encapsulate a wide range of therapeutic agents.^14^

In the context of cardiovascular disease, dendrimers have shown great potential for targeted drug delivery to specific sites of cardiac fibrosis. Their surface chemistry can be easily modified to attach targeting ligands, such as antibodies or peptides, enabling specific recognition and binding to diseased tissues or cells. Functionalizing dendrimers with ligands or antibodies specific to fibrotic tissue markers can enhance their binding and uptake by fibroblasts and myofibroblasts, improving therapeutic outcomes.^45^ Additionally, dendrimers can be engineered to release therapeutic agents in response to specific triggers, such as pH or enzyme activity, enabling site‐specific drug release within fibrotic regions.^33^

A notable study by Smith et al. demonstrated the potential of dendrimers in treating cardiovascular disease by delivering statins, a class of drugs used to lower cholesterol levels and inhibit atherosclerotic plaque formation. The study utilized polyamidoamine (PAMAM) dendrimers functionalized with folic acid as a targeting ligand for enhanced delivery to activated endothelial cells in atherosclerotic plaques. Conjugation of folic acid enabled specific binding of dendrimers to folate receptors overexpressed on activated endothelial cells leading to selective accumulation and drug release at the plaque sites. This targeted approach reduced systemic statin exposure, minimizing side effects while maximizing therapeutic efficacy. In addition, the study highlighted dendrimers as efficient carriers for site‐specific drug delivery in cardiovascular disease treatment.

Another study by Li et al. (2017) utilized mesenchymal stem cell‐derived exosomes encapsulated within dendrimers to treat cardiac fibrosis. The dendrimer‐based nanotherapeutic approach allowed for the targeted delivery of exosomes to fibrotic cardiac tissue, promoting regenerative processes and attenuating fibrosis progression. The study demonstrated the potential of dendrimers as carriers for therapeutic cargoes, highlighting their ability to enhance the therapeutic effects of stem cell‐derived exosomes in cardiac fibrosis treatment.

In addition to drug delivery, dendrimers have shown promise in other therapeutic applications including gene therapy, diagnostics, and imaging. Their well‐defined structure and highly branched architecture allow for efficient encapsulation and protection of nucleic acids, such as DNA or siRNA, facilitating their delivery to target cells for gene modulation. Furthermore, the multivalent nature of dendrimers enables the attachment of imaging agents, such as fluorophores or nanoparticles, facilitating their use as contrast agents for various imaging modalities.

In summary, dendrimers offer a tailored and versatile platform for targeted drug delivery in cardiovascular disease treatment including cardiac fibrosis. Their precise structure, functionalization ability, and triggered‐release capabilities make them promising nanocarriers for delivering therapeutic agents specifically to fibrotic tissues. In addition, ongoing research and development in this area hold great potential for advancing the field of nanomedicine and improving patient outcomes.

Micelles are self‐assembling nanoparticles composed of amphiphilic molecules in hydrophilic and hydrophobic regions. In an aqueous environment, these molecules spontaneously form nanoscale structures with a hydrophobic core and a hydrophilic shell, creating an ideal environment for encapsulating and delivering hydrophobic drugs.^46^ Like previously discussed nanocarriers, their unique structure offers several advantages as drug delivery carriers including improved solubility, enhanced stability, prolonged circulation time, and controlled drug release.^34^

A study by Oishi et al. highlighted the potential of micelles in cardiovascular disease treatment by delivering anti‐inflammatory drugs to inhibit the progression of atherosclerosis.^47^ In this study, micelles composed of a biodegradable polymer called poly(ethylene glycol)‐poly(lactic acid) (PEG‐PLA) were loaded with dexamethasone, a potent anti‐inflammatory agent.^47^ The micelles demonstrated excellent drug‐loading capacity and stability, effectively encapsulating and protecting dexamethasone from degradation. The micelles were specifically designed to target activated endothelial cells in atherosclerotic plaques by functionalizing the micelle surface with antibodies against intercellular adhesion molecule‐1 (ICAM‐1), a cell adhesion molecule overexpressed in inflamed endothelium. This targeted approach facilitated the selective accumulation and release of dexamethasone at the site of inflammation, effectively reducing inflammation and inhibiting the progression of atherosclerosis.^47^

Moreover, micelles have shown the potential to deliver anticancer drugs to treat cardiovascular diseases such as cardiac fibrosis. A study by Wang et al. developed micelles composed of poly(ethylene glycol)‐block‐poly(ε‐caprolactone) (PEG‐PCL) loaded with pirfenidone, an antifibrotic agent. The micelles exhibited excellent stability and controlled release of pirfenidone allowing for sustained drug delivery. In an in vivo mouse model of cardiac fibrosis, the micelles effectively accumulated and attenuated in the fibrotic myocardium, demonstrating their potential as therapeutic carriers for cardiac fibrosis treatment.

Like dendrimers, the use of micelles extends beyond drug delivery as they have also been employed in diagnostic imaging and theranostics. In addition, micelles can be functionalized with imaging agents, such as fluorescent dyes or magnetic nanoparticles, enabling their use as contrast agents for various imaging modalities including fluorescence and magnetic resonance imaging (MRI).^48^ This multifunctionality allows simultaneous diagnosis and targeted drug delivery, promoting personalized medicine approaches.

Overall, micelles are versatile nanoparticles with self‐assembling properties with great potential for drug delivery in cardiovascular diseases. The ability to encapsulate hydrophobic drugs, target specific cells or tissues, and combine diagnostics and therapeutics makes micelles a promising platform for personalized medicine.

While nanotechnology‐based drug delivery systems have shown great promise, several challenges must be addressed, such as ensuring their safety and biocompatibility, optimizing drug loading and release kinetics, and overcoming biological barriers for effective drug delivery.^45^ Nevertheless, with the rapid advances in nanotechnology and a deeper understanding of disease biology, the future of nanomedicine looks promising with potential applications spanning various disease areas including cardiac fibrosis.

## 7 NANOPARTICLES IN DIAGNOSTIC AND IMAGING APPLICATIONS

Nanoparticles have emerged as valuable tools not only in drug delivery but also in the field of diagnostics and imaging. Their unique properties and versatile nature make them well‐suited for various applications in this domain. Metallic nanoparticles, such as gold nanoparticles, have garnered significant attention in diagnostic imaging due to their exceptional optical properties.

Gold nanoparticles, for instance, exhibit strong and tunable absorption and scattering properties in the visible and near‐infrared regions, making them particularly useful for various imaging modalities.^49^ In optical imaging, gold nanoparticles can be utilized as contrast agents to enhance the visualization of biological structures and processes. By functionalizing their surfaces with targeting ligands or biomolecules, these nanoparticles can selectively bind to specific cellular or molecular targets, enabling the detection and imaging of disease‐related biomarkers.^49^

Furthermore, gold nanoparticles find applications in computed tomography (CT) imaging, where their high X‐ray attenuation properties facilitate the generation of detailed anatomical images.^50^ The ability of gold nanoparticles to absorb X‐rays effectively enhances the contrast in CT scans, enabling improved visualization of tissues and structures of interest. This has paved the way for their use in various preclinical and clinical imaging studies.

Photoacoustic imaging is another noteworthy application of metallic nanoparticles including gold nanoparticles. Photoacoustic imaging combines the benefits of optical and ultrasound imaging to produce high‐resolution images that penetrate deep into tissues. When exposed to laser pulses, gold nanoparticles can efficiently convert the absorbed light energy into acoustic waves, which can be detected and reconstructed into detailed images.^48^ This imaging modality holds great promise in various biomedical applications, including tumor imaging, vascular imaging, and functional brain imaging.

Polymeric nanoparticles, on the other hand, offer versatility in imaging applications. They can be tailored to encapsulate or conjugate with contrast agents, such as fluorescent dyes or magnetic nanoparticles, allowing for enhanced imaging sensitivity and specificity. For instance, polymeric nanoparticles loaded with near‐infrared fluorophores have been employed for fluorescence imaging.^51^ Incorporation of fluorescent dyes into the polymeric matrix enables the visualization of specific cellular targets or pathological processes with high sensitivity, contributing to early disease detection and monitoring.

Moreover, polymeric nanoparticles can be engineered to carry imaging probes and target‐specific ligands simultaneously, enabling multimodal imaging. By combining different imaging modalities, such as fluorescence imaging, magnetic resonance imaging (MRI), and positron emission tomography (PET), the strengths of each modality can be synergistically harnessed to provide complementary information and improve diagnostic accuracy.

In summary, metallic nanoparticles, particularly gold nanoparticles, and polymeric nanoparticles have proven to be valuable assets in the field of diagnostic imaging (Table 1). Their unique optical, magnetic, and structural properties make them versatile imaging agents. By harnessing their capabilities, researchers and clinicians can gain valuable insights into disease processes, visualize anatomical structures with enhanced precision, and develop innovative imaging approaches for improved diagnosis and patient care.

**TABLE 1:** Summary of nanoparticle types and their applications in cardiac fibrosis treatment.

Nanoparticle type	Therapeutic applications in cardiac fibrosis treatment
Liposomes	Targeted drug delivery to fibrotic cardiac tissue.
	Controlled release of encapsulated anti‐fibrotic drugs.
Polymeric nanoparticles	Encapsulation and targeted delivery of therapeutic agents.
	Modulation of fibrosis‐related signaling pathways.
Dendrimers	Targeted drug delivery to fibrotic cardiac tissue.
	Enhanced tissue penetration and cellular uptake.
Micelles	Encapsulation and controlled release of anti‐fibrotic drugs.
	Targeted drug delivery to fibrotic cardiac tissue.
Inorganic nanoparticles	Diagnostic imaging of cardiac fibrosis.
	Controlled drug release and theranostic applications.

## 8 ADVANCEMENTS IN ENHANCING BIOCOMPATIBILITY OF NANOPARTICLES

In recent years, considerable efforts have been dedicated to improving the biocompatibility of nanoparticles to ensure their safe utilization in biomedical applications. Enhancing biocompatibility involves various strategies with surface modifications crucial in mitigating potential adverse effects. One commonly employed approach is the addition of biocompatible coatings on the surface of nanoparticles. For example, polyethylene glycol (PEG) is frequently utilized as a coating material due to its excellent biocompatibility and ability to minimize interactions between nanoparticles and the immune system.^52^ The presence of PEG on the nanoparticle surface forms a protective barrier, reducing the immune recognition and prolonging circulation time in the body.

The selection of biocompatible materials is also pivotal in ensuring the biocompatibility of nanoparticles. Biodegradable polymers have gained prominence in nanoparticle synthesis due to their favorable biocompatible properties and ability to undergo gradual degradation within the body. PLGA, a widely utilized biodegradable polymer, offers excellent biocompatibility and biodegradability, making it a preferred choice for nanoparticle formulation.^53^, ^54^ The use of such biocompatible polymers contributes to the overall safety profile of nanoparticles as they minimize potential toxicity concerns associated with non‐biodegradable materials.

Optimizing the size and shape of nanoparticles is another critical aspect to enhance their biocompatibility. Studies have demonstrated that smaller nanoparticles generally exhibit improved biocompatibility and reduced toxicity compared to their larger counterparts.^52^ This size‐dependent effect is attributed to the increased surface area‐to‐volume ratio of smaller nanoparticles, facilitating efficient interactions with cells and reducing potential adverse effects. Additionally, controlling the shape of nanoparticles can influence their biocompatibility and cellular uptake. Nanoparticles with well‐defined shapes, such as spheres, rods, or disks, can offer improved biocompatibility by minimizing non‐specific interactions and enhancing the specific cellular targeting.

In recent years, researchers have also focused on developing “smart” nanoparticles that can respond to specific triggers in the biological environment, further enhancing their biocompatibility. These stimuli‐responsive nanoparticles can undergo size or surface property changes in response to various stimuli such as pH, temperature, enzymes, or specific biomolecules. Using stimuli‐responsive materials, nanoparticles can exhibit enhanced stability, controlled release of encapsulated drugs, and improved biocompatibility. For instance, pH‐responsive nanoparticles based on poly(ethylene glycol)‐poly(β–amino ester) copolymers can shrink or disassemble in the acidic tumor microenvironment, allowing better penetration into target tissues and controlled drug release.^31^

Furthermore, assessing the biocompatibility of nanoparticles involves comprehensively evaluating their potential cytotoxicity, immunogenicity, and long‐term effects on the body. Rigorous in vitro and in vivo studies are necessary to comprehend the interactions between nanoparticles and biological systems, enabling researchers to enhance nanoparticle formulations and reduce potential risks.

Collectively, recent efforts to improve the biocompatibility of nanoparticles have focused on surface modifications, material selection, size and shape optimization, and the development of stimuli‐responsive systems. These advancements aim to maximize nanoparticle safety and therapeutic potential in biomedical applications. By addressing biocompatibility considerations throughout the design and development stages, researchers can pave the way for the translation of nanoparticles into effective and safe clinical tools.

## 9 FUTURE DIRECTIONS

### 9.1 Enhancing therapeutic potential with nanoparticles

Due to their size (between 1 and 100 nm) and high surface‐to‐volume ratio, nanoparticles present a unique opportunity to advance medical treatment.^55^, ^56^ The small size and large surface area of these particles allow for biofunctionalization with biomolecules such as peptides, antibodies, metals, and polymers, thus enhancing cellular uptake and improving the targeting specificity.^57^ One major limitation of current cardiac fibrosis treatments is their lack of specific cellular targeting, reducing the therapeutic potential of otherwise effective drugs. However, nanoparticles can be engineered for greater selectivity, reducing toxic effects throughout the body. For example, anticancer drugs kill cancer cells and induce severe toxicity in healthy cells. Encapsulating these toxic drugs in nanoparticles can reduce their adverse effects on the body. At the same time, specificity can be improved with biomolecules, thus enhancing the cellular uptake of the targeted disease cells requiring therapy. In addition, various materials including liposomes,^58^ gold,^59^ silver iron oxide,^60^, ^61^ carbon nanomaterials,^62^ polymers,^63^ and nucleic acids,^64^ have been used in nanoparticle construction.

Current administration routes for nanoparticles predominantly rely on intravenous injection, a form of systemic delivery. While this is preferable for conditions requiring immediate relief, for progressive diseases such as cardiac fibrosis, systemic delivery of treatments may not always be effective. Despite their potential for specificity, Nanoparticles might not reach the diseased area in sufficient quantities due to fast clearance through the liver and kidneys, the organs first encountered by the bloodstream. Additionally, the liver readily absorbs lipophilic drugs and nanoparticles, limiting their circulation throughout the body; therefore, research on the localized delivery of therapeutic nanoparticles is gaining increasing interest.

### 9.2 Tissue engineering and nanoparticles

The integration of nanoparticles in tissue engineering is under investigation to direct the benefits of nanoparticle treatments to the site of action.^65^, ^66^ In cardiac tissue engineering, nanomaterials have been found to aid in the regeneration of cells including embryonic stem cells, mesenchymal stem cells, and induced pluripotent stem cells.^67^, ^68^ The use of patches, polymers, and hydrogels engineered with biomaterials and developed with 3D printing is under investigation.^69^ Injection of hydrogels directly into the heart has also been studied as this promotes treatment directly to the site of injury making them less invasive and clinically useful. While modification of these engineered bioactive molecules like peptides, growth factors, and gene modifiers can be challenging due to their susceptibility to physiological degradation, and conditions, nanomaterials have shown potential in stabilizing these molecules. The mechanical and electrical properties of nanoparticles also contribute to the biochemical signaling of bioactive molecules in cardiac tissues.^70^

#### 9.2.1 The potential of carbon nanomaterials

Discovered only within the last 4 decades, carbon nanomaterials have emerged as a promising tool in cardiac tissue engineering due to their unique properties. These nanomaterials, typically less than 100 nm and predominantly composed of carbon atoms, are classified into graphene, carbon nanotubes, and fullerene.^71^ Their appeal lies in their electrical conductivity, mechanical stiffness and strength, and surface modification abilities. The electrical conductivity of carbon nanomaterials is particularly advantageous for cardiomyocytes, cells responsible for heart contraction. This conductivity encourages the alignment of these cells in a single direction, thereby improving their contractility and more accurately replicating the function of cardiac tissue.^70^

**FIGURE 4:**
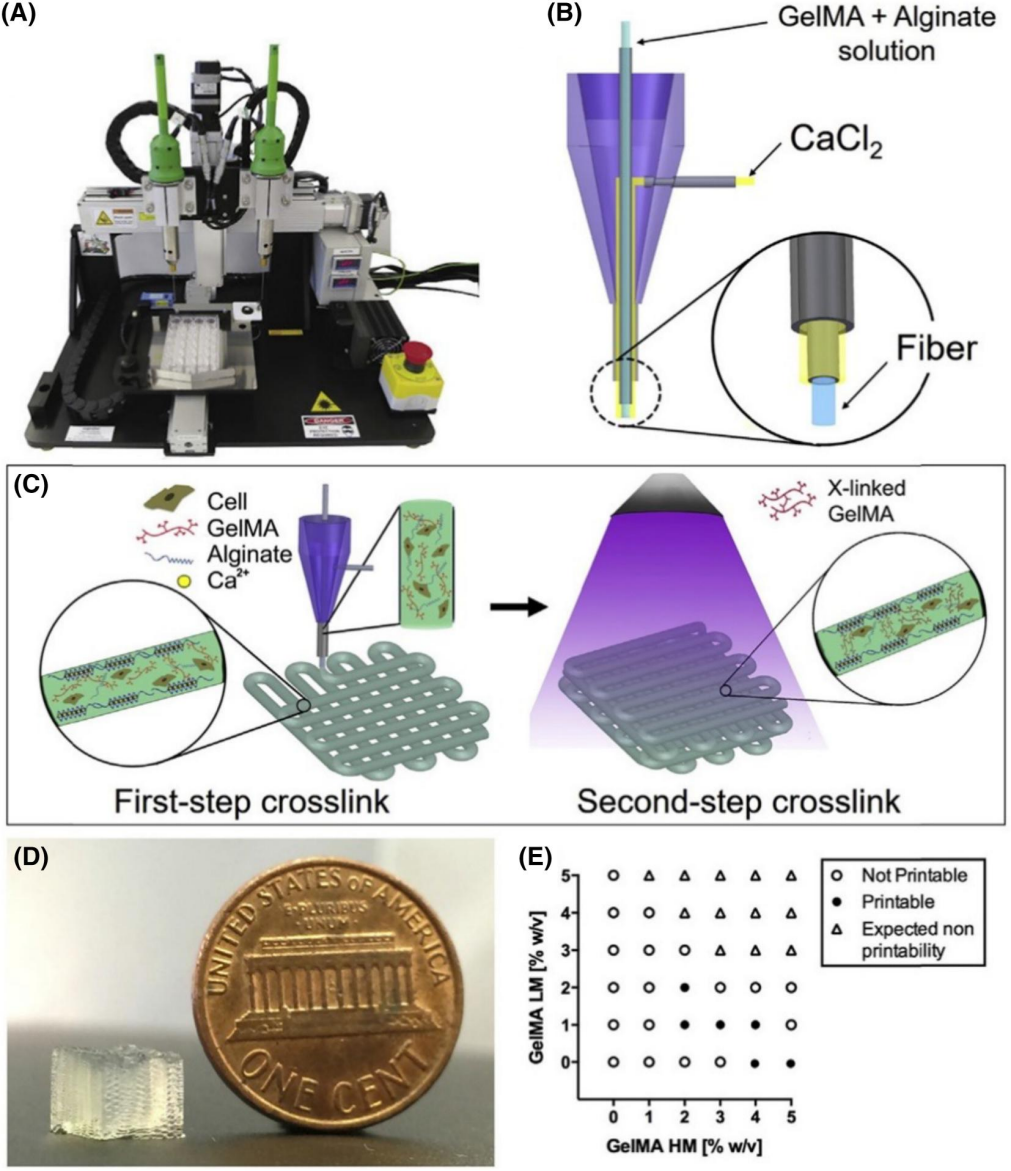
(A) Photograph of an organovo novogen MWX bioprinter. (B) Schematic of the coaxial needle where the bioink is delivered from the core and the ionic crosslinking CaCl_2_ solution is sheathed on the side. (C) Schematic diagrams showing the two‐step crosslinking process, where the alginate component is first physically crosslinked by the CaCl_2_ followed by chemical crosslinking of the GelMA component using UV illumination. (D) Photograph of a bioprinted cubic microfibrous scaffold (6‐mm edge length). (E) Bioink optimization where conditions of printability and non‐printability for different concentrations of GelMA‐HM and GelMA‐LM (with a constant alginate concentration of 4 w/4%) were analyzed. Reproduced with permission.^72^ Copyright 2016, Elsevier.

The anisotropic effect of carbon nanoparticles was investigated by Zhang et al. utilizing “heart on a chip” 3D printed materials to guide the formation of endothelial myocardium having contractile capabilities.^72^ The approach employed gelatin ink composed of gelatin methacryloyl (GelMA), alginate, and Irgracure crosslinker to create a microfibrous scaffold for growing tissue (Figure 4).^73^ Myoblasts were fabricated into cardiac contractile tissues by electrophoretically, aligning carbon nanotubes (CNTs) in the GelMA. The unidirectional alignment of CNT with an electric field developed a material with increased conductivity, promoting the expression of contractile proteins of the myoblasts. The researchers took advantage of the electrical properties of CNTs, allowing the fibers to demonstrate greater contractility compared to hydrogels absent of carbon nanotubes. Various studies have shown that using carbon nanoparticles in cardiac tissue engineering influences stem cell differentiation and cardiomyocyte phenotype in hydrogels,^74^ enhances synchronized contractions,^75^ improves cellular functions of specific cell types,^76^ and affects cellular conductivity, factors that are important for the regeneration of functional cardiac tissue.

Notably, the preparation methods of the materials used also play a significant role in the function of the nanoparticle.^77^ The placement of incorporation of carbon nanoparticles within hydrogel fibers can affect the directionality, elongation, and contractile protein expression.^78^ For example, incorporating CNTs coaxially with polymer fibers within a scaffold increases the elongation of cells and expression of α‐actinin and troponin compared to cells where CNTs were randomly distributed throughout the hydrogel.^79^ CNTs have also been shown to improve electrical conductivity and toughness when incorporated with electrospinning, enhancing cell viability and synchronic contractile behavior. Several studies using CNT hydrogels showed not only did carbon improve the electrical conductivity of the hydrogel but in animal models improved the cardiac function after infarction, especially when compared to hydrogels absent of carbon nanomaterials^57^, ^80^, ^81^, ^82^, ^83^, ^84^, ^85^ The versatility of tissue engineering also allows for the integration of growth factor, and when combined with graphene oxide to deliver vascular endothelial growth factor, which ultimately improves contractile performance, and tissue vascularization.^86^

#### 9.2.2 Magnetic nanoparticles for targeted therapy

Interestingly, magnetic nanoparticles are also becoming increasingly investigated since their integration allows for specific targeting of the disease site with ex vivo magnets. In one example, cardiosphere‐derived stem cells labeled with magnetic ferumoxytol nanoparticles demonstrated the ability to be targeted within coronary arteries in a rat model post‐myocardial infarction. This allowed the introduction of stem cell retention within an injured artery allowing for engraftment.^87^ Iron oxide magnetic nanoparticles conjugated with signal regulatory protein alpha antibody were also used to guide tissue scaffolds with magnets, demonstrating the ability to manipulate human cardiomyocytes.^88^ Iron oxide nanoparticle integration within tissue scaffolds allows for a guided direction to injured tissue, thus positively impacting cellular signaling and cellular phenotype, inducing enhanced targeted therapeutic effects.^89^ Carbon and magnetic nanoparticles present a unique opportunity to develop localized delivery approaches. In addition, tissue engineering applications can help mediate the limitations of systemic delivery approaches.

### 9.3 Epigenetic regulation

Epigenetic changes are considered a hallmark of heart failure, thus regulating the epigenome is a promising new avenue for treating cardiac diseases like fibrosis.^90^ Epigenetic modifications act by regulating the transcription observed in cardiac failure phenotypes. Those changes include post‐translational modifications (PTMs) of histone tails, DNA methylation, and less frequent 3D chromatin structure changes. Enzymatic chromatin remodelers are responsible for regulating the addition or removal of modifications to chromatin. These enzymes respond to genetic or environmental stress and work together to modify chromatin, ultimately leading to transcriptional changes observed in cardiac diseases. The most studied and well‐known inhibitors of epigenetic changes are histone deacetylases (HDAC) and bromodomain extra terminal inhibitors (BET). Epigenetic regulation of cardiac fibrosis is only recently being investigated. However, genetic regulation has been shown to improve and alleviate cardiac failure after fibrotic progression. It has been demonstrated that epigenetic regulators can modulate cardiac fibroblast proliferation, myofibroblast activation, and fibroblast senescence, critical factors in the development and progression of cardiac fibrosis.

Histone deacetylase (HDAC) inhibitors have demonstrated the ability to suppress cardiac fibroblast proliferation and alleviate fibrotic remodeling after cardiac injury. The mechanism of action of class I HDAC inhibitors to stop the proliferation of cardiac fibroblasts involves the induction of genes encoding cyclin‐dependent kinase inhibitors p15 and p57, eventually blocking downstream genes that promote cell cycle proliferation.^91^, ^92^ Similarly, fibroblast transition into myofibroblast is the hallmark of collagen deposition within the extracellular matrix due to the overexpression of contractile proteins like alpha‐SMA as well as matricellular ECM proteins including thrombospondin‐1 and ‐2, osteopontin, SPARC, periostin, tenascin‐C, and CCN2.^93^ The dense packing of collagen provides scar tissue formation that stiffens the surrounding tissue following cardiac injury furthering cardiac dysfunction. However, epigenetic modulation of myofibroblast transition has focused on the gene expression of alpha‐SMA. Trichostatin A, another class I HDAC inhibitor, was shown to reduce the activation of serine/threonine kinases and thereby block alpha‐SMA expression of lung fibroblasts.^94^ Alpha‐SMA expression was reversed in cardiac fibroblasts after administration of MGCD0103 (HDAC inhibitor).^95^ BET protein inhibitors have demonstrated potential as a treatment for cardiac fibrosis.^96^ ^,^ ^97^ Bromodomain‐containing protein 4 (BRD4), part of the acetyl‐histone binding protein family, has emerged as a critical regulator of cardiac fibrosis activation through the TGF‐beta pathway. BRD4 is upregulated in cardiac hypertrophy and thus plays a crucial role in stress response during cardiac disease. Due to BRD4's role in regulating the activation of cardiac fibrosis through TGF‐b signaling, its inhibition has been shown to alleviate cardiac fibrosis.^98^ ^,^ ^99^

Recent evidence has suggested that non‐coding RNAs (ncRNA) are involved in cardiac fibrosis development through post‐transcriptional regulation of gene expression. NcRNA is mostly microRNA (miRNA), long non‐coding RNA (lncRNA), and circular RNA (cirRNA) that function biologically without protein‐coding abilities. miRNA function depends on its complementary mRNA binding site. It can inhibit protein translation or mediate the post‐transcriptional gene silencing by targeting mRNA degradation and can either promote or inhibit fibrotic development.^100^ For example, Tao et al. demonstrated that miRNA‐369‐5p promotes the proliferation of fibroblasts through modulation of DNMT3A methylation which inhibits the Patched 1 pathway. In contrast, Yang et al. showed miRNA‐489 alleviated fibrosis by inhibiting fibroblast activation and proliferation by regulating HDAC2.^53^, ^101^

Epigenetic regulation thus offers promising pathways for targeting and possibly reversing the course of cardiac fibrosis by modifying the action of gene transcription without changing the genome. Understanding the mechanisms, interactions, and genes regulating cardiac scar formation is necessary to utilize epigenetic regulators to treat cardiac fibrosis. The difficulty in treating cardiac fibrosis and cardiac disease is after the development of scar tissue has formed. Thus understanding the processes and regulators involved will help facilitate optimized nanoparticle formulation and selection of gene regulators to treat better and eventually halt and prevent scar formation. Many drugs and nucleotide‐based molecules are not stable when given freely; they require a delivery vehicle that will maintain stability, increase circulation throughout the body, target the disease area, and nanoparticles can do this. While the results are promising, future research on epigenetic regulation using nanoparticles should look to understand the physiochemical interactions of genetic regulators and biomaterials chosen to deliver them. The genetic mechanisms of cardiac fibrosis are not completely understood. Therefore, future work utilizing epigenetic regulation should also aim to understand the genes involved in this process. As we uncover more about the nuances of cardiac fibrosis pathophysiology and the intricacies of nanoparticle behavior, we gain more opportunities to tailor treatments. This includes not only the nanoparticles' design but also the materials used and their preparation methods. Our journey toward effective, specific, and targeted cardiac fibrosis therapies has just begun. The promising potentials of carbon and magnetic particles, as well as gene regulation, warrant further exploration.

## 10 CONCLUSIONS

Cardiac fibrosis poses an evident and substantial health burden on a global scale, necessitating the development of effective therapeutic interventions. While impactful research has been conducted to understand and alleviate cardiac fibrosis, much work is still needed. The mechanisms and underlying causes are still not fully understood. However, further understanding of cardiac fibrosis development will bring potential new targets to stop and reverse its progression. Nanotechnology is an emerging field of research that uniquely allows functionalization, surface modification, and encapsulation of various materials, making it a promising strategy for addressing the complexities of cardiac fibrosis.

Nanoparticles have revolutionized drug delivery systems, offering unparalleled advantages in enhancing treatments' efficacy and therapeutic effects. Among the current delivery methods being investigated, liposomes, polymeric nanoparticles, dendrimers, and micelles have emerged as promising nanocarriers. Liposomes, composed of phospholipid bilayers, provide versatility in encapsulating hydrophilic and hydrophobic drugs, improving their stability and bioavailability. Polymeric nanoparticles, on the other hand, offer controlled and sustained drug release minimizing toxicity and enhancing therapeutic efficacy. With their highly branched structures, Dendrimers allow precise control over size, surface functionality, and drug‐loading capacity, enabling targeted and controlled release. Micelles, formed by the self‐assembly of amphiphilic molecules, provide efficient solubilization and delivery of hydrophobic drugs. These nanoparticle‐based drug delivery systems offer improved drug solubility, bioavailability, and targeted delivery, enhancing therapeutic outcomes. By harnessing the unique properties of nanoparticles, researchers aim to overcome the challenges associated with conventional drug delivery methods and pave the way for more effective and personalized medicine. Through the design and development of nanomaterials such as dendrimers, micelles, and polymeric nanoparticles, targeted drug delivery, gene therapy, and tissue engineering have shown remarkable potential in mitigating the detrimental effects of cardiac fibrosis and related cardiovascular diseases. These innovative approaches open the door to more specialized medicine by offering precise and controlled drug release, enhanced therapeutic efficacy, and improved patient outcomes.

Furthermore, continued research and investment in nanomedicine are crucial for unlocking its full potential. Long‐term studies and clinical trials are needed to evaluate the safety and efficacy of nanomedicines in diverse patient populations. Moreover, exploring novel applications such as combination therapies or the integration of nanotechnology with other emerging fields like artificial intelligence or gene editing holds promise for further advancements in the field.

While significant progress has been made in nanomedicine, challenges persist that need to be addressed to successfully translate nanotechnology‐based therapies into clinical practice. Safety, biocompatibility, regulatory considerations, and scalability of the manufacturing process require concerted efforts and collaboration. By addressing these challenges and continuing to invest in research and development, nanotechnology has the potential to revolutionize healthcare, offering innovative and effective solutions for the diagnosis, treatment, and prevention of diseases.

By harnessing the potential of nanotechnology and leveraging its unique properties, we can envision a future where cardiac fibrosis is managed more effectively, improving the quality of life for patients worldwide. The journey toward personalized, precise, and targeted therapies for cardiac fibrosis continues. With ongoing advancements in nanotechnology, we are on the cusp of transformative breakthroughs that will reshape the landscape of cardiovascular medicine.

In conclusion, nanotechnology provides hope in the fight against cardiac fibrosis, offering innovative solutions to address the global health burden posed by this devastating condition. Continuous research, collaboration, and a commitment to patient‐centered approaches can pave the way for a future where cardiac fibrosis is effectively managed and patients can lead healthier lives.

## CONFLICT OF INTEREST STATEMENT

The authors declare no conflict of interest.
